# Association between body mass index and mortality in the Korean elderly: A nationwide cohort study

**DOI:** 10.1371/journal.pone.0207508

**Published:** 2018-11-16

**Authors:** Seung-Hyun Lee, Do-Hoon Kim, Joo-Hyun Park, Shinhye Kim, Moonyoung Choi, Hyonchong Kim, Da Eun Seul, Soo Gyeong Park, Jin-Hyung Jung, Kyungdo Han, Young-Gyu Park

**Affiliations:** 1 Department of Family Medicine, Korea University, College of Medicine, Seoul, Republic of Korea; 2 Department of Biostatistics, Catholic University College of Medicine, Seoul, Republic of Korea; Tulane University School of Public Health and Tropical Medicine, UNITED STATES

## Abstract

The objective of this study was to investigate the relationship between body mass index (BMI) and mortality in the elderly. This study was a nation-wide population-based retrospective cohort study of the National Health Insurance System-Senior Database (NHIS-SD). In this study, a total of 75,856 subjects were identified and selected from among 251,593 individuals aged ≥ 65 years who underwent health screening at least once between 2009 and 2012 and who had no history of diabetes, cardiovascular disease, stroke, cancer, or chronic obstructive pulmonary disease (COPD). The subjects of this study were followed-up until 2013 to identify the total mortality and the cause-specific mortality of 6 groups divided according to BMI. The hazard ratio (HR) by reference group (23 ≤ BMI < 25 kg/m^2^) of each group was calculated. A significant increase in the HR with a decreased BMI was observed in the group with a BMI < 23 kg/m^2^, whereas the HR in the group with a BMI ≥ 25 kg/m^2^ was not significantly different than that of the reference group (23 ≤ BMI < 25 kg/m^2^). This pattern was also seen in the subgroup analyses in relation to age, smoking history, alcohol use, exercise level, and socioeconomic status. In this study, we found that a low BMI was a risk factor for death in the elderly and that no significant difference in mortality was seen in the elderly with a BMI of 25 or over. In terms of an optimal BMI in the elderly, it is important to maintain an appropriately healthy range of BMI with the aim of preventing weight loss.

## Introduction

It is well known that obesity is closely related to the development of chronic pathologies such as hypertension, diabetes, and cardiovascular diseases [1, 2]. The degree of obesity is often measured using the body mass index (BMI) [3, 4]. Previous studies have reported that the relationship between mortality and BMI is not linear but that it is a U-shaped or J-shaped curve [5–14], indicating that those with a low or a high BMI have higher mortality in contrast to those with a normal BMI. It has also been reported that low body weight is associated with higher mortality [11, 12, 15, 16].

The relationship between BMI and mortality varies among different age groups and ethnicities [5, 9, 10, 14, 17, 18]. In 2003, the World Health Organization (WHO) recognized that Asians have a higher risk of type 2 diabetes, hypertension, and dyslipidemia at a lower BMI than Europeans. Based on these findings, the WHO suggested that a lower optimal BMI range is needed for Asian-origin populations [3]. Previous studies have also shown that the relationship between BMI and mortality differs between younger adult populations and elderly populations.

Given these findings, it may be difficult to uniformly apply BMI criteria to specific elderly populations. Furthermore, the Korean population may demonstrate a different association between mortality and BMI in contrast to that observed in the Western population. Therefore, we opted to conduct this nationwide population-based retrospective cohort study with the aim of investigating the relationship between BMI and mortality in the elderly Korean population.

## Materials and methods

### Setting and participants

The National Health Insurance System-Senior Database (NHIS-SD) is a subsample of the National Health Insurance Service Claim Database (NHIS-CD). NHIS-SD consists of a 10% sample of randomly selected elderly individuals aged over 60 years who were living in Korea at the end of 2002. The database contains demographic and household income data (including socioeconomic factors), medical records, health screening check-up results, and questionnaires [19]. The NHIS Claim Database can be considered representative of the Korean population because almost all Koreans are covered by the National Health Insurance Service. By using the NHIS database, we were able to obtain follow-up mortality data.

Among the cohort, we selected 251,593 people aged ≥ 65 years who were found to have had a waist circumference measurement taken at least once during health screening between 2009 and 2012. In Korea, the age at which long-term care insurance benefits are generally applied is over 65. Therefore, we defined individuals aged over 65 years old at the time of the study cohort entry as elderly.

After applying the exclusion criteria, a final study population was selected. In order to minimize reverse causality and the effect of underlying disease on mortality, patients who had indicated a history of diabetes, cardiovascular disease, or stroke in the questionnaire were excluded from this study. In addition, those who were diagnosed with diabetes, cancer, myocardial infarction, stroke, or COPD were also excluded from this study. As this was a retrospective study, the need for individual consent was waived. All data were anonymous and de-personalized. This study was approved by the Institutional Review Board of Korea University Ansan Hospital.

Between 2009 and 2012, we identified 251,593 people aged ≥ 65 years who had undergone health screening at least once; of these, 2,178 were excluded owing to missing data. Another 53,390 individuals were excluded due to their self-reported history of cardiovascular disease, stroke, and diabetes (as indicated in the questionnaires), and 120,169 were further excluded based on a clinical diagnosis of myocardial infarction, stroke, COPD, and diabetes according to disease codes, yielding a total of 75,856 study subjects. These subjects were followed-up until 2013 to identify the total mortality and the cause-specific mortality rates. The mean follow-up period was 3.65 years. During this time, there were 3,717 deaths, of which 1,489 (40.1%) were due to cancer and 728 (19.6%) were due to cardiovascular disease.

### Determinants of disease and demographic factors

BMI was calculated by dividing the weight (kg) by the square of the height (m^2^). The participants were divided into 6 groups based on their BMI: < 18.5 kg/m^2^; 18.5 ≤ BMI < 23 kg/m^2^; 23 ≤ BMI < 25 kg/m^2^; 25 ≤ BMI < 27.5 kg/m^2^; 27.5 ≤ BMI < 30 kg/m^2^; and > 30.0 kg/m^2^. Height and waist circumference were measured in centimeters, and body weight was measured in kilograms.

We divided participants according to these categories in order to classify possible subgroups based on generally accepted obesity criteria for the Korean population and determine the optimal weight range. During this process, it was determined that the BMI criteria for Asians are different from those of Caucasians.

A detailed history of smoking, alcohol use, and exercise level was collected using the questionnaires. The populations were further stratified according to age (< 75 years, ≥ 75 years), exercise level (strenuous exercise at least 3 days a week or moderate exercise for at least 5 days a week), smoking status (current smokers, non-smokers), and alcohol use (alcohol users, non-users). The socioeconomic status (SES) of the subjects was evaluated, and the subjects were allocated into 2 groups (lower 20% plus Medicaid, the others). The criteria for evaluating the socioeconomic status of the subjects were based on the average monthly insurance premiums imposed by the Korean National Health Insurance Corporation (NHIC), which are determined based on poverty level and monthly household income in Korea.

The medical history of the subjects was collected based on the ICD-10 diagnoses, pharmacy codes, prescribed medications, and previous medical histories. ICD-10 codes were used to define the cause of death, such as cardiovascular disease (I00-I99) and cancer (C00-D48).

### Clinical outcomes

The clinical outcomes of this study included the total all-cause mortality and the cause of death (cancer, myocardial infarction, stroke). Total mortality was further stratified according to age, exercise level, smoking status, and alcohol drinking.

### Statistical analysis

The mortality for each BMI group was adjusted for variables including age, exercise level, smoking status, alcohol use, and SES. The Cox proportional hazard regression model was used to calculate the hazard ratio (HR). The log-log survival (LLS) plot for each outcome is plotted to determine the cox proportion assumption, and the curves are parallel to each BMI level, so the proportional assumption is satisfied (S1 Fig). The HR and 95% confidence interval were calculated for each BMI group and compared to the reference group (23 ≤ BMI < 25 kg/ m^2^). In this elderly sample, there was a real possibility of reverse causality, so in an effort to reduce this possibility, we conducted a detailed subgroup analysis, excluding those who died in the first year of cohort entry. Older people who died within one year of cohort enrollment are more likely to have died of previous illnesses that could directly affect BMI. All statistical analyses were performed using SAS version 9.3 software. All tests were two-tailed, and p-values < 0.05 were considered statistically significant.

## Results

### Study subjects

The characteristics of the subjects are summarized in Table 1. There were significantly fewer males (42.3%) than females. The mean age of the subjects was 72.7 ± 4.7 years (males, 72.2 ± 4.4 years; females, 73.1 ± 4.9 years). The proportion of those aged ≥ 75 years was 26.4%. The proportion of current smokers was 24.9%. The proportion of those who used alcohol was 26.0%. In terms of exercise, 16.8% of the subjects were categorized into exercise groups. The proportion of those with a low SES was 23.1%.

**Table 1:** Participants characteristics.

	Total	Male	Female
Characteristic	N = 75856	N = 32065	N = 43791
Age (yr)	72.7 ± 4.69	72.22 ± 4.35	73.05 ± 4.9
Age ≥ 75yr, n (%)	20035 (26.4)	7255 (22.6)	12780 (29.2)
Current smoker, n (%)	18852 (24.9)	17531 (54.7)	1321 (3.0)
Drinker, n (%)	19737 (26.0)	16383 (51.1)	3354 (7.7)
Exercising, n (%)^a^	12751 (16.8)	7441 (23.2)	5310 (12.1)
Low SES, n (%)^b^	17523 (23.1)	7690 (24.0)	9833 (22.5)
Deaths, n (%)	3717 (4.9)	2158 (6.7)	1559 (3.6)
Deaths due to cancer (%)	1489 (2.0)	1003 (3.1)	486 (1.1)
Deaths due to CVD (%)	728 (1.0)	376 (1.2)	352 (0.8)
Height (cm)	156.0 ± 9.1	164.2 ± 5.8	150.1 ± 5.8
Weight (kg)	57.6 ± 9.9	62.8 ± 9.0	53.8 ± 8.6
BMI (kg/m^2^)	23.6 ± 3.1	23.3 ± 2.9	23.8 ± 3.3
Waist circumference (cm)	82.1 ± 8.3	83.8 ± 8.0	80.9 ± 8.4
Serum glucose (mg/dL)	98.2 ± 18.8	99.6 ± 20.6	97.3 ± 17.4
SBP (mmHg)	131.1 ± 16.5	131.5 ± 16.5	130.9 ± 16.6
DBP (mmHg)	78.7 ± 10.1	79.1 ± 10.2	78.4 ± 10.1
Serum cholesterol (mg/dL)	201.7 ± 38.2	192.3 ± 35.8	208.5 ± 38.4
Follow Up Duration (yr)	3.7 ± 1.0	3.7 ± 1.0	3.6 ± 1.0

Data was presented as percentages if dichotomous and mean ± SD if continuous.

^a^Intensive exercise 3 or more times per a week, or moderate exercise 5 or more times per a week

^b^Lower 30% of socioeconomic status

SD = standard deviation; SES = socioeconomic status; CVD = cardiovascular disease; BMI = body mass index; SBP = systolic blood pressure; DBP = diastolic blood pressure.

The characteristics of the subjects in different BMI groups are shown in S1 Table. The mean age was higher in the lower BMI groups, which was observed in both males and females. The smoking rate was higher in the lower BMI groups. The blood glucose level, cholesterol level, and blood pressure tended to increase along with BMI.

### Association between body mass index and all-cause mortality

The relationship between BMI and all-cause mortality is shown in Table 2. A correction for age and sex revealed an inverse J-shaped association between BMI and all-cause mortality. The HR was lowest in the moderately obese group (27.5 ≤ BMI < 30 kg/m^2^), but this was not statistically significant (HR 0.903; 95% CI 0.756–1.077). The highest mortality was observed in the group with the lowest BMI (< 18.5 kg/m^2^). In the group with a BMI < 23.0 kg/m^2^, there was a significant increase in the HR in accordance with a decrease in BMI, whereas the HR in the group with a BMI ≥ 25.0 kg/m^2^ did not differ significantly from that in the reference group (23.0 ≤ BMI < 25 kg/m^2^).

**Table 2:** Association between body mass index and all-cause and cause-specific mortality.

		Total				Lag period 1 years^a^			
		n = 75627				n = 74916			
BMI		Crude rate	Hazard ratio (CI)			Crude rate	Hazard ratio (CI)		
(kg/m^2^)		Per 1000 person-years	Model 1	Model 2	Model 3	Per 1000 person-years	Model 1	Model 2	Model 3
< 18.5	All-Cause Mortality (Total)	39.71	2.6(2.31,2.93)	2.51(2.23,2.84)	2.58(2.24,2.97)	41.23	2.46(2.15,2.82)	2.37(2.07,2.72)	2.55(2.18,3.00)
18.5 ≤ BMI < 23		16.66	1.42(1.30,1.55)	1.40(1.28,1.52)	1.41(1.29,1.55)	18.67	1.42(1.29,1.57)	1.40(1.27,1.54)	1.44(1.30,1.60)
23 ≤ BMI < 25		10.00	1(Ref.)	1(Ref.)	1(Ref.)	11.26	1(Ref.)	1(Ref.)	1(Ref.)
25 ≤ BMI < 27.5		9.39	1.02(0.91,1.14)	1.03(0.92,1.15)	1.02(0.91,1.14)	10.57	1.02(0.90,1.15)	1.03(0.91,1.16)	1.00(0.89,1.14)
27.5 ≤ BMI < 30		7.71	0.92(0.78,1.09)	0.93(0.79,1.10)	0.92(0.77,1.09)	8.97	0.95(0.79,1.14)	0.96(0.80,1.16)	0.92(0.76,1.11)
≥ 30		8.73	1.15(0.89,1.50)	1.15(0.89,1.50)	1.12(0.85,1.47)	9.85	1.16(0.87,1.55)	1.16(0.87,1.54)	1.08(0.80,1.45)
< 18.5	All-Cause Mortality (Male)	46.56	2.64(2.25,3.10)	2.52(2.15,2.96)	2.76(2.27,3.35)	48.77	2.47(2.06,2.96)	2.35(1.96,2.82)	2.68(2.15,3.33)
18.5 ≤ BMI < 23		22.18	1.44(1.29,1.61)	1.40(1.26,1.57)	1.46(1.29,1.65)	25.39	1.47(1.30,1.66)	1.43(1.27,1.62)	1.52(1.33,1.73)
23 ≤ BMI < 25		13.99	1(Ref.)	1(Ref.)	1(Ref.)	15.68	1(Ref.)	1(Ref.)	1(Ref.)
25 ≤ BMI < 27.5		12.73	0.96(0.83,1.11)	0.97(0.84,1.12)	0.94(0.81,1.10)	14.51	0.98(0.83,1.15)	0.99(0.84,1.16)	0.95(0.80,1.12)
27.5 ≤ BMI < 30		11.93	0.92(0.72,1.17)	0.93(0.73,1.19)	0.88(0.69,1.13)	13.84	0.95(0.73,1.23)	0.96(0.74,1.26)	0.89(0.68,1.17)
≥ 30		12.97	1.01(0.63,1.62)	1.01(0.63,1.63)	0.93(0.57,1.51)	17.90	1.24(0.77,2.00)	1.25(0.78,2.01)	1.10(0.67,1.79)
< 18.5	All-Cause Mortality (Female)	34.34	2.40(2.00,2.89)	2.38(1.98,2.86)	2.28(1.85,2.81)	35.27	2.32(1.88,2.86)	2.29(1.86,2.83)	2.31(1.82,2.93)
18.5 ≤ BMI < 23		11.97	1.38(1.19,1.58)	1.37(1.19,1.58)	1.34(1.16,1.56)	12.95	1.34(1.14,1.56)	1.33(1.14,1.56)	1.34(1.13,1.58)
23 ≤ BMI < 25		6.88	1(Ref.)	1(Ref.)	1(Ref.)	7.81	1(Ref.)	1(Ref.)	1(Ref.)
25 ≤ BMI < 27.5		7.12	1.10(0.92,1.30)	1.11(0.93,1.31)	1.12(0.94,1.33)	7.89	1.07(0.88,1.29)	1.07(0.89,1.3)	1.07(0.88,1.30)
27.5 ≤ BMI < 30		5.84	0.93(0.73,1.19)	0.94(0.74,1.2)	0.97(0.75,1.25)	6.80	0.95(0.73,1.24)	0.96(0.74,1.25)	0.96(0.73,1.26)
≥ 30		7.67	1.27(0.92,1.75)	1.27(0.92,1.75)	1.32(0.94,1.86)	7.86	1.13(0.78,1.63)	1.13(0.78,1.64)	1.12(0.76,1.66)
< 18.5	Deaths due to cancer	10.10	1.78(1.44,2.21)	1.70(1.37,2.10)	1.89(1.47,2.42)	10.89	1.66(1.31,2.12)	1.58(1.24,2.01)	1.76(1.33,2.32)
18.5 ≤ BMI < 23		6.68	1.33(1.17,1.52)	1.31(1.14,1.49)	1.37(1.19,1.58)	7.66	1.32(1.15,1.53)	1.29(1.12,1.49)	1.36(1.16,1.59)
23 ≤ BMI < 25		4.54	1(Ref.)	1(Ref.)	1(Ref.)	5.25	1(Ref.)	1(Ref.)	1(Ref.)
25 ≤ BMI < 27.5		4.02	0.95(0.80,1.12)	0.96(0.81,1.14)	0.93(0.78,1.11)	4.64	0.95(0.79,1.14)	0.96(0.80,1.15)	0.93(0.77,1.12)
27.5 ≤ BMI < 30		3.43	0.91(0.71,1.18)	0.93(0.72,1.20)	0.87(0.66,1.13)	4.19	0.96(0.74,1.27)	0.98(0.75,1.29)	0.92(0.69,1.22)
≥ 30		4.15	1.28(0.87,1.87)	1.28(0.87,1.88)	1.15(0.77,1.72)	4.53	1.21(0.79,1.86)	1.21(0.79,1.86)	1.09(0.70,1.71)
< 18.5	Deaths due to cardiovascular diseases	7.32	2.13(1.62,2.81)	2.06(1.57,2.71)	2.19(1.59,3.02)	7.22	2.14(1.55,2.95)	2.05(1.49,2.84)	2.37(1.63,3.45)
18.5 ≤ BMI < 23		3.13	1.26(1.04,1.53)	1.24(1.02,1.50)	1.27(1.03,1.57)	3.51	1.40(1.12,1.76)	1.38(1.1,1.72)	1.47(1.16,1.87)
23 ≤ BMI < 25		2.08	1(Ref.)	1(Ref.)	1(Ref.)	2.09	1(Ref.)	1(Ref.)	1(Ref.)
25 ≤ BMI < 27.5		1.98	1.03(0.81,1.32)	1.04(0.82,1.33)	1.02(0.80,1.31)	2.11	1.09(0.82,1.44)	1.1(0.83,1.46)	1.05(0.79,1.4)
27.5 ≤ BMI < 30		1.74	0.98(0.68,1.40)	0.99(0.69,1.42)	0.95(0.65,1.38)	1.68	0.93(0.61,1.43)	0.94(0.62,1.45)	0.86(0.55,1.34)
≥ 30		1.43	0.86(0.45,1.63)	0.85(0.45,1.62)	0.80(0.41,1.56)	1.77	1.05(0.53,2.08)	1.05(0.53,2.07)	0.90(0.45,1.84)

^a^excluding those who died within one year to reduce the likelihood of reverse causality

Model 1 adjusted for age, sex

Model 2 adjusted for age, sex, smoking status, drinking status, exercise level, and SES

Model 3 adjusted for age, sex, smoking status, drinking status, exercise level, SES, and waist circumference.

Models for all-cause mortality stratified by gender were adjusted for age, smoking status, drinking status, exercise level, SES, and waist circumference.

In both sexes, the HR increased with a decrease in BMI for those with a BMI < 23.0 kg/m^2^ (Table 2). The HR in the group with a BMI ≥ 25.0 kg/m^2^ did not differ significantly from that in the reference group (23.0 ≤ BMI < 25 kg/m^2^). We also attempted a subgroup analysis to reduce the likelihood of reverse causality, excluding those who died within one year. The results showed similar pattern to the results of all participant analysis (Table 2).

This pattern was consistently seen in the subgroup analyses in relation to age, smoking history, alcohol use, exercise level, and SES (Table 3). In both sexes, the HR increased with a decrease in BMI for those with a BMI of < 23.0 kg/m^2^. The HR in the group with a BMI ≥ 25.0 kg/m^2^ did not differ significantly from that in the reference group (23.0 ≤ BMI < 25 kg/m^2^) in all subgroups. After stratifying for SES, the middle and high SES groups showed a similar increase in the HR with a low BMI and a less clear difference in the HR with increasing BMI. However, there was a slight increase in the HR with increasing BMI in the low SES group, but this was not statistically significant.

**Table 3:** Subgroup analysis association between body mass index and all-cause mortality.

BMI (kg/m^2^)	Crude rate, Per 1000 person-years	Model1	Model2	Crude rate, Per 1000 person-years	Model1	Model2
		Hazard ratio(95% CI)			Hazard ratio(95% CI)	
		**60 ≤ Age < 75**			**Age ≥ 75**	
< 18.5	14.7	2.909(2.402,3.524)	2.739(2.26,3.319)	41.8	2.281(1.947,2.671)	2.253(1.924,2.639)
18.5 ≤ BMI < 23	7.1	1.491(1.32,1.684)	1.457(1.29,1.645)	20.9	1.368(1.206,1.551)	1.351(1.191,1.532)
23 ≤ BMI < 25	4.5	1(Ref.)	1(Ref.)	13.6	1(Ref.)	1(Ref.)
25 ≤ BMI < 27.5	4.7	1.078(0.931,1.249)	1.086(0.938,1.258)	12.0	0.945(0.795,1.123)	0.951(0.8,1.13)
27.5 ≤ BMI < 30	4.0	1.005(0.81,1.248)	1.01(0.813,1.254)	9.2	0.8(0.606,1.056)	0.808(0.612,1.066)
≥ 30	4.5	1.262(0.907,1.757)	1.248(0.896,1.736)	10.8	0.993(0.644,1.531)	0.999(0.648,1.539)
		**Non-smoker**			**Current smoker**	
< 18.5	23.7	2.36(2.048,2.72)	2.341(2.032,2.698)	36.2	2.286(1.814,2.88)	2.273(1.803,2.865)
18.5 ≤ BMI < 23	9.6	1.422(1.286,1.571)	1.411(1.276,1.559)	18.3	1.262(1.054,1.511)	1.253(1.047,1.5)
23 ≤ BMI < 25	5.7	1(Ref.)	1(Ref.)	13.6	1(Ref.)	1(Ref.)
25 ≤ BMI < 27.5	5.7	1.081(0.956,1.224)	1.081(0.956,1.224)	11.3	0.867(0.665,1.13)	0.864(0.662,1.126)
27.5 ≤ BMI < 30	4.7	0.953(0.792,1.147)	0.952(0.792,1.146)	10.6	0.846(0.541,1.321)	0.854(0.546,1.334)
≥ 30	5.2	1.146(0.864,1.52)	1.139(0.858,1.511)	16.1	1.272(0.625,2.588)	1.252(0.615,2.549)
		**Non-drinker**			**Drinker**	
< 18.5	27.4	2.539(2.205,2.925)	2.492(2.164,2.87)	23.3	2.486(1.965,3.144)	2.328(1.84,2.946)
18.5 ≤ BMI < 23	10.3	1.416(1.273,1.575)	1.399(1.258,1.556)	12.8	1.487(1.276,1.735)	1.434(1.23,1.673)
23 ≤ BMI < 25	6.1	1(Ref.)	1(Ref.)	7.8	1(Ref.)	1(Ref.)
25 ≤ BMI < 27.5	5.9	1.058(0.925,1.209)	1.062(0.929,1.214)	6.9	0.932(0.761,1.143)	0.947(0.773,1.161)
27.5 ≤ BMI < 30	4.8	0.934(0.765,1.141)	0.941(0.771,1.149)	6.1	0.856(0.618,1.186)	0.865(0.624,1.199)
≥ 30	5.7	1.216(0.911,1.625)	1.211(0.907,1.618)	5.9	0.864(0.458,1.629)	0.879(0.466,1.657)
		**No exercise**			**Exercises**	
< 18.5	21.0	2.704(1.888,3.873)	2.631(1.837,3.769)	27.1	2.524(2.221,2.869)	2.47(2.173,2.807)
18.5 ≤ BMI < 23	7.9	1.329(1.054,1.676)	1.308(1.037,1.65)	11.6	1.445(1.314,1.588)	1.428(1.299,1.569)
23 ≤ BMI < 25	5.5	1(Ref.)	1(Ref.)	6.8	1(Ref.)	1(Ref.)
25 ≤ BMI < 27.5	5.0	0.975(0.732,1.299)	0.974(0.731,1.297)	6.4	1.027(0.91,1.159)	1.036(0.918,1.17)
27.5 ≤ BMI < 30	5.2	1.058(0.698,1.604)	1.056(0.696,1.6)	5.0	0.887(0.736,1.069)	0.896(0.743,1.08)
≥ 30	6.0	1.29(0.629,2.647)	1.307(0.637,2.683)	5.7	1.109(0.836,1.469)	1.112(0.839,1.474)
		**Middle-to-high SES**			**Low SES**	
< 18.5	25.2	2.487(2.161,2.863)	2.405(2.09,2.768)	30.2	2.764(2.191,3.488)	2.685(2.128,3.388)
18.5 ≤ BMI < 23	10.6	1.439(1.3,1.592)	1.416(1.279,1.568)	12.5	1.422(1.198,1.689)	1.4(1.178,1.662)
23 ≤ BMI < 25	6.3	1(Ref.)	1(Ref.)	7.4	1(Ref.)	1(Ref.)
25 ≤ BMI < 27.5	5.9	1.011(0.888,1.151)	1.018(0.894,1.159)	7.2	1.04(0.835,1.295)	1.053(0.845,1.311)
27.5 ≤ BMI < 30	4.7	0.893(0.732,1.089)	0.897(0.735,1.094)	6.1	0.969(0.697,1.348)	0.984(0.707,1.368)
≥ 30	5.1	1.044(0.762,1.43)	1.044(0.762,1.431)	7.7	1.394(0.867,2.242)	1.386(0.862,2.229)

Model 1 adjusted for age and gender, but only gender in subgroup analysis for aging stratification

Model 2 adjusted for age, sex, smoking status, drinking status, exercise level, and SES

A variable for each subgroup classification was not adapted as a control in model 2. For example, smoking variable was not adjusted in model 2 for subgroup analysis in accordance with smoking status. The same modeling approach applies to the other subgroups as well.

Additionally, S1 Fig is the Kaplan-Meier survival curve showing the differences in all-cause mortality, cancer mortality, and CVD mortality by BMI category group.

### Association between body mass index and cause-specific mortality

The relationship between BMI and cause-specific mortality is shown in Table 2. The relationship between BMI and cardiovascular-related death was not different from that between BMI and all-cause mortality. Cardiovascular-related mortality had the lowest HR in the slightly obese group (27.5 ≤ BMI < 30 kg/m^2^), but this was not statistically significant. However, the HR was higher with lower BMI in those with a BMI < 23 kg/m^2^.

A similar result was seen for BMI and cancer-related death, though the inverse J-shape was weak in contrast to that of the cardiovascular disease model. The HR was higher with lower BMI in those with a BMI < 23 kg/m^2^, whereas there was no significant difference in the HR with increased BMI in those with a BMI ≥ 25 kg/m^2^.

## Discussion

In this retrospective cohort study of the elderly in Korea, the relationship between all-cause mortality and cause-specific mortality and BMI was analyzed. In general, the relationship between all-cause mortality and cardiovascular and cancer-related mortality and BMI showed an inverse J-shaped distribution, in both the male and female subgroups. In contrast to the reference group (23 ≤ BMI < 25 kg/m^2^), the group with a BMI < 23 kg/m^2^ showed increased mortality with decreasing BMI, whereas there was no significant relationship between BMI and mortality in those with a BMI ≥ 25 kg/m^2^. This pattern was unchanged in further stratified analyses based on age, smoking status, alcohol use, exercise level, and SES.

A recent Korean study based on the NHIS national standard cohort data of the Korean adult population [12], as well as other previous studies on the total adult population, have generally showed a U-shaped distribution of mortality for increasing BMI, with increased mortality in the groups with either a high or a low BMI. However, in the present study, the relationship between mortality and BMI had an inverse J-shaped distribution. This suggests that age is a factor affecting the relationship between BMI and mortality. Previous population-based studies from Korea and overseas have either shown less of a rise in mortality with increasing BMI in the elderly in contrast to a younger population or unchanged mortality with increasing BMI [5, 14, 17, 18]. These studies, together with the current study, all suggest that the effect of obesity on the mortality rate is reduced in the elderly population, with a higher mortality in those with a low BMI. Whereas previous studies showed higher cardiovascular-related mortality with increasing BMI in the general adult population [15], the cardiovascular-related mortality did not significantly increase with increasing BMI in the elderly in this study. Interestingly, while a BMI increase of > 23 kg/m^2^ was not associated with a clear increase in the mortality rate among the elderly, the mortality rate was significantly higher in those with a lower BMI. The risks associated with a low BMI, as described in previous studies [11, 12, 14], tend to be poorly evaluated. Hong et al. have suggested that this may be due to a relatively small number of individuals with a low BMI in American and European populations and possibly to reverse causality theory [14]. Reverse causality refers to the phenomenon whereby the result has an effect on the cause via a different pathway. Therefore, a reverse causality theory of low BMI and mortality suggests that low BMI is not an independent risk factor of death, but rather that other factors which have an adverse effect on general health also contribute to a low BMI. An example of this is the association between smoking, underlying disease, and BMI [20]. However, previous large-scale studies in the U.S. [21] and studies on the Korean population that corrected for smoking status and excluded patients with chronic diseases to eliminate reverse causality still showed a significant association between low BMI and mortality [11, 12, 14]. In this study, we sought to eliminate reverse causality by excluding those with underlying disease such as cancer, cardiovascular disease, stroke, diabetes, and respiratory disease and by correcting for smoking status. Nevertheless, the results of this study still carry the potential for reverse causality, which must be considered when interpreting the results.

The higher mortality in elderly subjects with a lower BMI can be explained in two ways. First, low BMI is associated with sarcopenia. The relationship between sarcopenia and mortality has been reported in many previous studies in which it was demonstrated that sarcopenia increases the total mortality and risk of hospital readmission [22, 23], with a documented reduction in BMI in patients with sarcopenia [24] and higher rates of sarcopenia in low-weight elderly subjects [22]. Thus, elderly subjects with a lower BMI have more sarcopenia than those with a higher BMI, which may be a risk factor for higher mortality in elderly subjects with various comorbidities. Future studies on mortality and muscle mass, as measured by limb muscle circumference, will further elucidate this hypothesis. Second, low BMI is associated with malnutrition. Previous studies on hospital inpatients showed that those with a poor nutritional status had a lower BMI and higher mortality [25, 26]. Another long-term study on old patients showed that nutritional status and BMI were associated with mortality [27]. Therefore, elderly people with a lower BMI are more likely to have poor nutrition than those with a higher BMI, which may influence the total mortality.

The effect of SES on the relationship between BMI and mortality in elderly is another interesting factor. In this study, the subjects were divided into either a low SES group (lower 20% plus Medicaid) or a middle-to-high SES group. A greater increase in mortality in those with a BMI < 23 kg/m^2^ was observed in the lower SES group, along with a higher mortality in those with a BMI ≥ 27.5 kg/m^2^, although this did not have statistical significance. The results of previous studies have suggested that low SES populations are prone to either obesity or decreased body weight, and their results have demonstrated a significant relationship between SES and mortality, which may be attributable to lower educational status, poor health-related behaviors, and limitations in the capabilities of local community health systems and accessibility [28–31].

This study has a number of strengths. First, the NHIS elderly cohort data included various factors, including BMI, mortality, sex, age, smoking status, exercise level, alcohol use, and SES, to enable a stratified analysis and more accurate estimations. Second, this was a large-scale study with > 70,000 elderly subjects whose data were used to calculate a valid association between BMI and mortality. The elderly cohort database is a research resource designed to support the elderly, with information regarding risk factors and prognosis of geriatric diseases. Approximately 550,000 people, about 10% of the 5.5 million Korean elderly people (aged 60 years or older) who had maintained their health insurance and medical care status at the end of December 2002, were enrolled in this cohort. Based on the public health report of the Korean Center for Diseases Control (KCDC), the prevalence of obesity among older adults was 34.2% in 2012, which is similar to the obesity prevalence estimated by the elderly cohort. Third, to reduce the possibility of reverse causality, the effects of important health behaviors on mortality, including smoking, alcohol and exercise, were considered together, and subjects with a medical history of cancer, cardiovascular disease, cerebrovascular disease, diabetes, and respiratory disease were excluded, which still resulted in a significant relationship between low BMI and high mortality.

This study has also several limitations. First, this study is based on NHIS data and only included subjects who had undergone health screening. Since those who undergo health screening at least once every two years may have a greater interest in their health and wellbeing in contrast to those who do not and may be more likely to have a healthy lifestyle, this may have introduced a selection bias. Second, the proportion of very obese subjects with a BMI greater than 30 kg/m^2^ was only 2.5% of the elderly population, which made stratification of this group difficult. Future studies with a greater number of subjects will allow for a subgroup analysis of very obese subjects, a more meaningful analysis of the relationship between an increased BMI and mortality, and calculation of the optimal and acceptable BMI range for the elderly. Third, the mortality rate was corrected for waist circumference measurement in this study, but specific measures of body composition, such as the fat mass, muscle mass, and body fat component could not be considered. Fourth, since the mean follow-up duration in this study was only 3.7 years, there is the possibility that too few deaths were observed considering the high level of longevity among the Korean elderly, which undermines the study results. In order to compensate for this, it will be necessary to increase the follow-up period in subsequent studies and to include other outcomes such as morbidities, activities of daily living (ADL), and performance status.

There were two remarkable findings in this study. First, the effect of a high BMI on increased mortality was less in the elderly than that in the total adult population. Previous studies on the total adult population suggested a relatively small optimal BMI range, whereby any increase in the BMI above this optimal BMI range resulted in increased mortality. However, there was no clear increase in mortality in the elderly with increasing BMI above the BMI range of 23 ≤ BMI < 30 kg/m^2^, which made it difficult to estimate the optimal elderly BMI range. While the results from this study alone cannot be used to definitively conclude that a high BMI is not associated with increased mortality in the elderly, it may be that the effect of an increased BMI on the mortality is less significant in the elderly in contrast to that in the total adult population. The groups with 18.5 ≤ BMI < 23 kg/m^2^ (which corresponded to the optimum BMI range of 18.5 ≤ BMI < 23 kg/m^2^ for Asians, as suggested by the WHO) [3] all had significantly higher HRs in contrast to the reference BMI (23 ≤ BMI < 25 kg/m^2^) group, which suggests that the WHO BMI criteria may not be applicable to all elderly populations worldwide. Additionally, the results of this study suggest that a low BMI may be a more prominent risk factor for future mortality in the elderly, rather than mild obesity. Hence, the maintenance of an appropriately healthy range of BMI with a focus on preventing weight loss should be prioritized in the elderly.

## Supporting information

S1 Table Additive baseline characteristics according to body mass index category. (DOCX)

S1 Fig The log-log survival (LLS) plot for all-cause mortality, cancer mortality, and CVD mortality to determine the cox proportion assumption. the proportional assumption is satisfied because the curves are parallel to each BMI level.
(DOCX)
