# Supplementary material for: Association between body mass index and mortality in the Korean elderly: A nationwide cohort study
Source: PLoS One. 2018 Nov 16;13(11):e0207508. doi: 10.1371/journal.pone.0207508 (PMC6239300; doi:10.1371/journal.pone.0207508)
Supplement: S1 Table — (DOCX) [file pone.0207508.s001.docx]

**Supplementary table**

**S1 Table. Additive Baseline Characteristics according to Body Mass Index Category.**

|  | BMI(kg/m**^2^)** | | | | | |
| --- | --- | --- | --- | --- | --- | --- |
|  | <18.5 | 18.5 - 23 | 23 - 25 | 25 - 27.5 | 27.5 - 30 | ≥30 |
|  | Total | | | | | |
| Age, yr, mean ± SD | 75.54±6.35 | 73.18±5.01 | 72.3±4.33 | 72±4.11 | 71.95±3.95 | 71.94±3.9 |
| Age ≥ 75yr, n (%) | 1566(46.63) | 8952(30.25) | 4507(23.51) | 3336(21.08) | 1183(20.51) | 381(19.86) |
| Smoking, n (%) | 729(21.71) | 4768(16.11) | 2123(11.08) | 1317(8.32) | 375(6.5) | 93(4.85) |
| Drinker, n (%) | 828(24.66) | 8167(27.6) | 5149(26.86) | 4011(25.35) | 1254(21.74) | 303(15.8) |
| Exercise, n (%) | 2972(88.51) | 24873(84.06) | 15640(81.6) | 12956(81.87) | 4809(83.37) | 1681(87.64) |
| Low SES, n (%) | 814(24.24) | 7099(23.99) | 4304(22.46) | 3537(22.35) | 1276(22.12) | 441(22.99) |
| Height, cm, mean ± SD | 155.41±9.8 | 156.19±9.29 | 156.59±8.9 | 156.07±8.92 | 154.73±8.49 | 152.88±8.1 |
| Weight, kg, mean ± SD | 42.16±5.9 | 51.93±6.9 | 59.02±6.82 | 63.74±7.42 | 68.41±7.6 | 74.22±8.65 |
| Waist circumference, cm, mean ± SD | 69.63±6.53 | 77.32±6.1 | 83.25±5.62 | 87.31±5.76 | 91.43±6.28 | 96.25±7.33 |
| Serum glucose, mg/dL, mean ± SD | 96.27±19.62 | 96.92±18.96 | 98.61±19 | 99.73±18.75 | 100.81±18.72 | 102.91±22.48 |
| SBP, mmHg, mean ± SD | 126.28±17.75 | 129.22±16.68 | 131.63±16.13 | 133.3±16.02 | 134.54±16.04 | 136.44±16.13 |
| DBP, mmHg, mean ± SD | 76.5±10.61 | 77.76±10.15 | 78.83±9.99 | 79.77±9.95 | 80.38±10.01 | 81.66±10.19 |
| Serum cholesterol, mg/dL, mean ± SD | 189.52±36.99 | 198.72±37.8 | 203.32±38.16 | 205.04±37.97 | 207.45±38.11 | 209.11±39.83 |
| Deaths, n | 456(13.58) | 1802(6.09) | 707(3.69) | 549(3.47) | 164(2.84) | 61(3.18) |
| Deaths due to cancer, n | 116(3.45) | 723(2.44) | 321(1.67) | 235(1.48) | 73(1.27) | 29(1.51) |
| Deaths due to CVD, n | 84(2.5) | 339(1.15) | 147(0.77) | 116(0.73) | 37(0.64) | 10(0.52) |
| Duration, yr, mean ± SD | 3.42±1.16 | 3.66±1.01 | 3.69±0.97 | 3.69±0.96 | 3.69±0.95 | 3.64±0.99 |
|  | Male | | | | | |
| Age, yr, mean ± SD | 73.95±5.27 | 72.61±4.61 | 71.92±4.13 | 71.55±3.84 | 71.5±3.63 | 71.48±3.71 |
| Age ≥ 75yr, n (%) | 525(35.81) | 3508(25.85) | 1717(20.39) | 1093(17.19) | 295(16.59) | 55(14.4) |
| Smoking, n (%) | 629(42.91) | 4324(31.86) | 1930(22.92) | 1170(18.4) | 308(17.32) | 71(18.59) |
| Drinker, n (%) | 672(45.84) | 6974(51.39) | 4338(51.53) | 3249(51.1) | 937(52.7) | 189(49.48) |
| Exercise, n (%) | 1226(83.63) | 10649(78.46) | 6318(75.04) | 4743(74.6) | 1335(75.08) | 290(75.92) |
| Low SES, n (%) | 318(21.69) | 3330(24.54) | 1981(23.53) | 1528(24.03) | 419(23.57) | 81(21.2) |
| Height, cm, mean ± SD | 163.93±5.96 | 163.97±5.9 | 164.31±5.73 | 164.53±5.67 | 164.41±5.7 | 164.28±5.86 |
| Weight, kg, mean ± SD | 47±4.23 | 57.09±5.29 | 64.8±4.77 | 70.55±5.22 | 76.88±5.58 | 84.69±8.31 |
| Waist circumference, cm, mean ± SD | 70.9±6.06 | 79.21±5.65 | 85.61±4.94 | 89.95±5.04 | 94.91±5.67 | 100.2±7.39 |
| Serum glucose, mg/dL, mean ± SD | 97.19±21.57 | 98.12±21.25 | 100.35±21.02 | 101.63±19.9 | 102.93±19.48 | 104.84±19.28 |
| SBP, mmHg, mean ± SD | 126.6±17.71 | 129.77±16.71 | 132.24±16.12 | 133.83±15.81 | 135.49±16.02 | 137.08±15.71 |
| DBP, mmHg, mean ± SD | 76.72±10.44 | 78.24±10.18 | 79.31±10.03 | 80.3±9.98 | 81.16±10.01 | 82.4±10.43 |
| Serum cholesterol, mg/dL, mean ± SD | 179.77±35.6 | 189.94±35.52 | 194.84±36.32 | 195.63±34.94 | 196.97±34.57 | 198.45±36.23 |
| Deaths, n | 235(16.03) | 1102(8.12) | 434(5.16) | 301(4.73) | 78(4.39) | 18(4.71) |
| Deaths due to cancer, n | 80(5.46) | 512(3.77) | 219(2.6) | 147(2.31) | 39(2.19) | 9(2.36) |
| Deaths due to CVD, n | 41(2.8) | 185(1.36) | 84(1) | 48(0.75) | 16(0.9) | 4(1.05) |
| Duration, yr, mean ± SD | 3.44±1.16 | 3.66±1.01 | 3.69±0.98 | 3.72±0.95 | 3.68±0.95 | 3.63±0.97 |
|  | Female | | | | | |
| Age, yr, mean ± SD | 76.78±6.82 | 73.66±5.28 | 72.6±4.45 | 72.3±4.25 | 72.15±4.06 | 72.06±3.94 |
| Age ≥ 75yr, n (%) | 1041(55.02) | 5444(33.98) | 2790(25.96) | 2243(23.69) | 888(22.26) | 326(21.22) |
| Smoking, n (%) | 100(5.29) | 444(2.77) | 193(1.8) | 147(1.55) | 67(1.68) | 22(1.43) |
| Drinker, n (%) | 156(8.25) | 1193(7.45) | 811(7.55) | 762(8.05) | 317(7.94) | 114(7.42) |
| Exercise, n (%) | 1746(92.28) | 14224(88.79) | 9322(86.73) | 8213(86.75) | 3474(87.07) | 1391(90.56) |
| Low SES, n (%) | 496(26.22) | 3769(23.53) | 2323(21.61) | 2009(21.22) | 857(21.48) | 360(23.44) |
| Height, cm, mean ± SD | 148.82±6.57 | 149.59±5.93 | 150.54±5.67 | 150.39±5.59 | 150.42±5.42 | 150.05±5.75 |
| Weight, kg, mean ± SD | 38.41±3.97 | 47.56±4.76 | 54.49±4.28 | 59.17±4.65 | 64.64±4.84 | 71.62±6.5 |
| Waist circumference, cm, mean ± SD | 68.65±6.72 | 75.72±6 | 81.39±5.43 | 85.54±5.53 | 89.88±5.9 | 95.26±6.97 |
| Serum glucose, mg/dL, mean ± SD | 95.55±17.93 | 95.89±16.72 | 97.25±17.13 | 98.45±17.82 | 99.87±18.3 | 102.43±23.19 |
| SBP, mmHg, mean ± SD | 126.03±17.79 | 128.76±16.64 | 131.14±16.13 | 132.94±16.15 | 134.12±16.03 | 136.28±16.23 |
| DBP, mmHg, mean ± SD | 76.33±10.73 | 77.35±10.1 | 78.46±9.95 | 79.43±9.92 | 80.03±9.99 | 81.47±10.12 |
| Serum cholesterol, mg/dL, mean ± SD | 197.07±36.28 | 206.15±38.07 | 209.96±38.26 | 211.36±38.61 | 212.11±38.69 | 211.76±40.26 |
| Deaths, n | 221(11.68) | 700(4.37) | 273(2.54) | 248(2.62) | 86(2.16) | 43(2.8) |
| Deaths due to cancer, n | 36(1.9) | 211(1.32) | 102(0.95) | 88(0.93) | 34(0.85) | 20(1.3) |
| Deaths due to CVD, n | 43(2.27) | 154(0.96) | 63(0.59) | 68(0.72) | 21(0.53) | 6(0.39) |
| Duration, yr, mean ± SD | 3.4±1.15 | 3.65±1.01 | 3.69±0.97 | 3.68±0.96 | 3.69±0.95 | 3.65±0.99 |

Data was presented as percentages if dichotomous and mean ± SD if continuous.

^a^Intensive exercise 3 or more times per a week, or moderate exercise 5 or more times per a week

^b^Lower 20% of socioeconomic status plus Medicaid

SD = standard deviation; SES = socioeconomic status; CVD = cardiovascular disease; BMI = body mass index; SBP = systolic blood pressure; DBP = diastolic blood pressure.
