# Supplementary material for: Association between body mass index and mortality in the Korean elderly: A nationwide cohort study
Source: PLoS One. 2018 Nov 16;13(11):e0207508. doi: 10.1371/journal.pone.0207508 (PMC6239300; doi:10.1371/journal.pone.0207508)

Supplementary Figure Legend

S1 Fig. The log-log survival (LLS) plot for all-cause mortality, cancer mortality, and CVD mortality to determine the cox proportion assumption. the proportional assumption is satisfied because the curves are parallel to each BMI level.


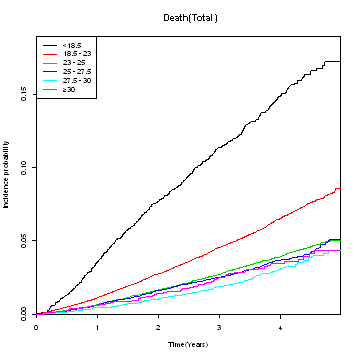

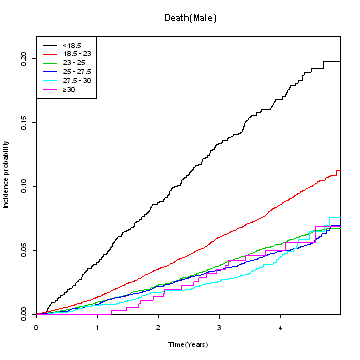

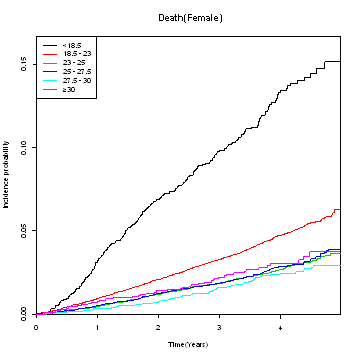


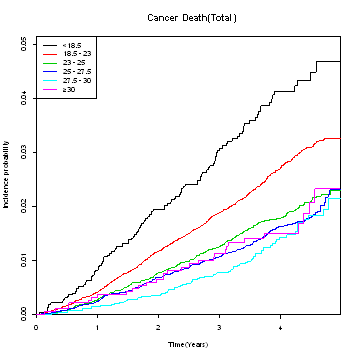

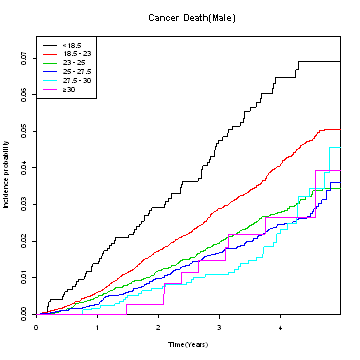

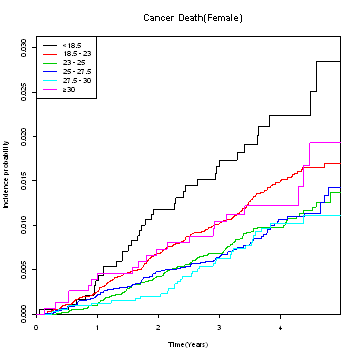


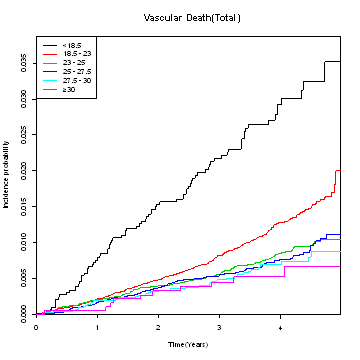

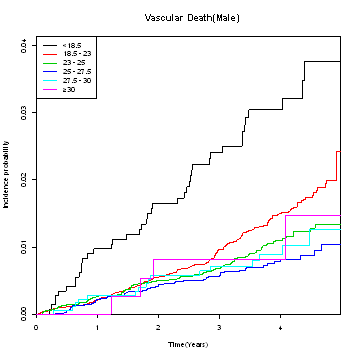

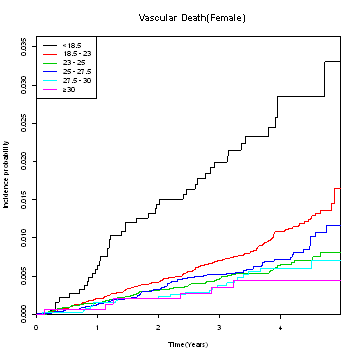

Supplement: S1 Fig — the proportional assumption is satisfied because the curves are parallel to each BMI level. (DOCX) [file pone.0207508.s002.docx]
